# Dynamical analysis of coronavirus disease with crowding effect, and vaccination: a study of third strain

**DOI:** 10.1007/s11071-021-07108-5

**Published:** 2022-01-04

**Authors:** Ali Raza, Muhammad Rafiq, Jan Awrejcewicz, Nauman Ahmed, Muhammad Mohsin

**Affiliations:** 1 Department of Mathematics, Government Maulana Zafar Ali Khan Graduate College Wazirabad, Punjab Higher Education Department (PHED), Lahore, 54000 Pakistan; 2 Department of Mathematics, Faculty of Sciences, University of Central Punjab, Lahore, 54500 Pakistan; 3 Department of Automation, Biomechanics and Mechatronics, Lodz University of Technology, 1/15 Stefanowskiego St., 90-924 Lodz, Poland; 4 Department of Mathematics and Statistics, The University of Lahore, Lahore, Pakistan; 5 Department of Mathematics, Technische Universitat Chemnitz, Chemnitz, Germany

**Keywords:** Coronavirus, Mathematical model, Reported cases, Strength number, Second derivative Lyapunov analysis, Numerical results

## Abstract

Countries affected by the coronavirus epidemic have reported many infected cases and deaths based on world health statistics. The crowding factor, which we named "crowding effects," plays a significant role in spreading the diseases. However, the introduction of vaccines marks a turning point in the rate of spread of coronavirus infections. Modeling both effects is vastly essential as it directly impacts the overall population of the studied region. To determine the peak of the infection curve by considering the third strain, we develop a mathematical model (susceptible–infected–vaccinated–recovered) with reported cases from August 01, 2021, till August 29, 2021. The nonlinear incidence rate with the inclusion of both effects is the best approach to analyze the dynamics. The model's positivity, boundedness, existence, uniqueness, and stability (local and global) are addressed with the help of a reproduction number. In addition, the strength number and second derivative Lyapunov analysis are examined, and the model was found to be asymptotically stable. The suggested parameters efficiently control the active cases of the third strain in Pakistan. It was shown that a systematic vaccination program regulates the infection rate. However, the crowding effect reduces the impact of vaccination. The present results show that the model can be applied to other countries' data to predict the infection rate.

## Introduction

The acronym SARS stands for severe acute respiratory syndrome. Initially, it was discovered in Asia in 2003. From Asia, it spreads to North and South America, as well as Europe. It was, however, contained in 2004. High fever, headache, body pains, diarrhea, dry cough, and pneumonia are symptoms of SARS caused by the coronavirus. The word corona means "crown." There is a small crown of spike proteins visible. The spike proteins bind to the ACE2 receptors on our respiratory system's cells. After the cells combine, the coronavirus injects its viral RNA into our cells, which is coded to produce more proteins and more virus cells. SARS cov2, also called COVID -19, is the cause of the current pandemic. It began in December 2019 in Wuhan, China. The virus was previously found in a wild animal, most likely a bat, and it was discovered in humans via tests conducted during a pneumonia outbreak in Wuhan. It then traveled all over the world, eventually reaching the USA on January 20, 2020. They can also be transmitted via air droplets and are capable of causing difficulties in breathing. Other symptoms can be mild or severe. Everyone is vulnerable, but it affects the elderly the most. People with underlying medical issues, such as cancer or obesity, are housed in senior living homes or rehab facilities. The immunocompromised individuals and those of similar nature are susceptible, and it is possible for those who recovered from the first infection to be infected again. The global death rate is low, yet there are still a lot of people dying. The USA has one of the highest infection rates in the world. On March 11, 2020, it was declared a pandemic with an R0 (reproduction number) of approximately 2.5. The number of fatalities per 100,000 persons in most countries affected by the pandemic is on the increase. Brazil has the highest mortality rate of 264.6 per 100,000, while Vietnam has the lowest death rate of 2.15 per 100,000. With 187.15 deaths per 100,000, the USA is in the lead. COVID -19 is a unique type of virus. The strain is SARS COV2, and symptoms occur two to fourteen days after exposure. As a result, it is easier to spread. COVID -19 has a higher death rate than the flu. It is more infectious. If it persists from weeks to months, one may lose the sense of taste or smell, resulting in long-term consequences such as lung problems. A polymerase chain reaction (PCR) test or an antigen test is available to figure out if one is infected or not. Antigen tests and PCR tests are two of the most common infection tests now available. The antigen test is quick and inexpensive to carry out; however, it is up to 20% inaccurate. The PCR test, on the other hand, is highly potent in terms of detection. It simply takes a few cells to detect the presence of coronavirus. The results can be obtained in a matter of hours. Depending on the type of PCR test one gets, a good PCR test can be 100 percent accurate. A nasopharyngeal swab is inserted into the nose and used to clean the area leading to the throat. It does not affect one's brain regardless of how much it appears to be. The swab is then rotated in each nostril for 15 s to ensure enough germs or bacteria are picked up. An antibody test can be used to determine if one has ever been infected with the coronavirus. Antibodies indicate that the immune system has successfully attacked the virus. It can tell whether or not a vaccine is effective. If, however, one is newly infected, a quarantine or simple isolation (up to ten days) with medical care is adequate for mild cases. For severe cases leading to difficulties in breathing or organ failure, treatment in the (intensive care unit of a) hospital is recommended. Finally, we discuss the prevention for which social distancing, uses of masks, and sanitizers are vital components. We should also wash our hands frequently and get vaccinated. A distance of six feet from sick persons or other individuals is recommended inside and out outside buildings. The importance of masks cannot be overstated. Even in vaccinated areas, cases of old and new strains (variants) have been recorded. But masks are not required outside unless it is windy or crowded places where the spread is likely to be faster. Facemasks prevent the disease from spreading through the air to other humans and objects we may contact. After contacting any surface, we must wash our hands for at least 20 s before touching our face, going to the restroom, or preparing meals. Hand sanitizers containing at least 60% alcohol can be used if we cannot wash our hands. On COVID -19 vaccination, the spike proteins on the virus hold on to ACE2 and force the RNA into our cells, causing more COVID -19 virus to be produced. This process causes other cells to be infected. Our body's genetic material (mRNA) directs the secretion of the same proteins, the T-cells, and the lymphocytes, to target viral RNA. As a result, our bodies combat the virus. Pfizer vaccines are for people aged 12 and up, while Johnson & Johnson, and Moderna are for people aged 18 and over. The vaccines were released in phases, with phase 1A targeting healthcare workers and elders living in communities, followed by others with a lower priority classification. We must be aware that some persons may experience allergic responses to these immunizations. It is not common, but it can happen. According to some conspiracy theorists, COVID does not exist. They believe it is a government myth designed to keep us under control. One theory has it that masks give us COVID because of our inner COVID. Furthermore, that the use of facemasks will kill us due to our CO2. If this is true, the doctors who have been standing in a surgery room for hours would have been long dead. Some people speak of herd immunity. Vaccines, on the other hand, provide herd immunity. Only the immuned people can prevent the virus from spreading [21].

### Literature review

Ahmed et al. proposed the SEQIIR model in 2021 to evaluate the evolution of COVID using ODEs and FDEs [1]. In 2021, Hassan et al. interpreted their investigation using the SIIR model to analyze the trends of the coronavirus in Texas, USA [2]. Alqarni et al. presented a DSIARB model to examine the complexities of the coronavirus in the Kingdom of Saudi Arabia in 2020 [3]. Savi et al. created a SEIRDC model to test the interplay of coronavirus in Brazil in 2020 [4]. Tiwari et al. noted in a SEIRD forecasting, COVID-19 outbreak dispersion under the influence of quarantine in India in 2020 [5]. In 2021, Warbhe et al. presented an S-I-R-M model to determine the COVID-related losses [6].

In 2021, Daniel investigated the SEIQCRW model on COVID diffusion with irregular factors of illness and the need for safety practices in Nigeria [7]. For estimation dynamics of the coronavirus disorder 2019 (COVID-19) outbreak, isolation security controls, Prathumwan et al. proposed a SLIQHR model in 2020 [8]. In 2021, Balike investigated the SEIHQR model for reducing the economic impacts of the COVID in the Congo [9]. In 2021, Raslan examined a SEHQIR model for COVID-19 infection projection in Egypt [10]. In 2020, Chen et al. proposed a BHRP model used to simulate the step in the process propagation of a new coronavirus [11].

In 2021, Sinaga et al. suggested an SEIR model in Indonesia and examined the coronavirus's structural analysis [12]. In 2020, Biswas et al. proposed using the SEAIQHR model to explore the COVID-19 contagion in India [13]. The use and misuse of mathematical modeling for communicable diseases management should be investigated, according to James et al. in 2021 [14]. In 2021, Ameen et al. proposed the use of SSLLIPD model to examine the kinetics of COVID-19 by applying a partial computational formula [15]. In estimating emerging coronavirus epidemiology, Uddin et al. proposed the SUQC model in 2020 [16]. In another development, Jiang et al. in 2020 studied a SEIIRMH model for determining the best SARS-CoV-2 removal plan in China, South Korea, and Italy [17]. In 2021, Kahn et al. used a model to guide COVID vaccination programs and testing procedures in nursing homes [18]. For the COVID -stability study employing fast-slow breakdown, Chen et al. developed a subtype of the SIR epidemic model in 2020 [19]. Staying at home, keeping distances, and early identification are the core points in controlling the spread of COVID -19, according to Kim et al. in 2020 [20]. Machado et al. studied the pandemic of coronavirus via complex systems that have characteristics that give rise to the emergence of rare and extreme events [28]. Rajagopal et al. investigated the fractional-order model to predict the dynamics of coronavirus outbreaks [29]. Quaranta et al. studied a multi-scale territorial analysis of the pandemic using various models and data-driven approaches in Italy [30]. Different types of dynamical analysis are studied to model coronavirus-like diseases as presented in [31–36]. Many mathematical models have been analyzed with the help of different strategies, as illustrated by authors in [37–42]. The stability analysis of COVID-19 model is studied under the properties of fractional calculus in [44].

The design of our paper is as follows. In Sect. 2, we discussed the mathematical model and performed its analysis. Then, in the subsections, positivity, boundedness, existence, and uniqueness are examined. In Sect. 3, parameter estimation is presented. Finally, in Sect. 4, the numerical results are outlined to analyze the dynamics of the virus graphically. We then discussed the local and global stability of the model and gave concluding remarks in the last section.

## Model formulation

**Fig. 1:**
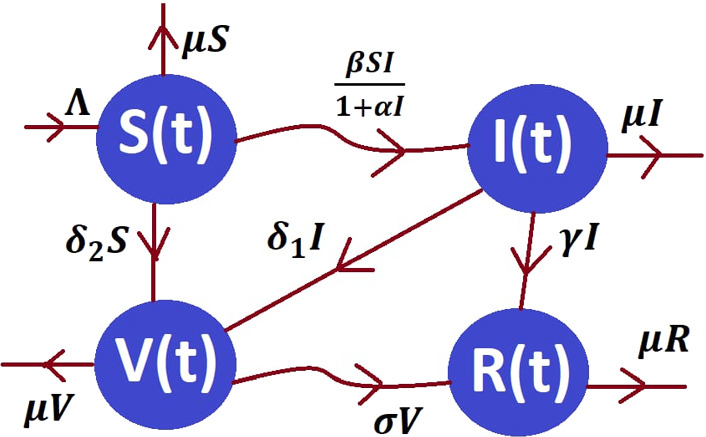
Flow map coronavirus model

By the theory of the population dynamics, the human population $N\left(t\right)$ is categorized into the four subpopulations like $S(t)$: (Susceptible population), $I(t)$: (Infected population), $V(t)$: (Vaccinated population), and $R(t)$: (Recovered or immune population). A dynamic of infection into the population is based on the law of mass action (the rate of change of reacting substance is directly proportional to the product of interacting substances) and nonlinear complex ordinary differential equations (ODEs). The transmission map of the model is shown in Fig. 1.

The physical relevance of parameters of the model is as follows: $\Lambda N$: (the recruitment rate of the population), $\beta I$: (the force of infection of virus), $\frac{1}{1+\alpha I}$: (the crowding effect of population on the virus), $\mu$: (the rate of mortality due to virus or natural of each subpopulation), ${\delta }_{1}$: (the rate at which infected population got vaccination during the period of quarantine, or isolation, etc.), ${\delta }_{2}$: (the rate at which susceptible population got vaccination under the program launched by World Health Organization (WHO)), $\gamma :$ (the rate at which infected population may recover due to its internal immunity and natural circumstances), and $\sigma :$ (the rate of doses in the population who recovered or got immune after vaccination). Deterministic modeling based on the following assumptions are as follows: Susceptible population directly vaccinated, considering the two types of immunity for an infected population like vaccination, and isolation or quarantine or natural immunity, and recovered population may not be infected again. Other types of interaction are ignored without loss of generality. The system of equations obtained from the transmission map of the virus is as follows:

1 $$ \begin{aligned} \frac{dS}{{dt}}  & =  \Lambda - \frac{\beta S\left( t \right)I\left( t \right)}{{1 + \alpha I\left( t \right)}} - \left( {\delta_{2} + \mu } \right)S\left( t \right), t \ge 0, \\ \frac{dI}{{dt}}  & =  \frac{\beta S\left( t \right)I\left( t \right)}{{1 + \alpha I\left( t \right)}} - \left( {\gamma + \delta_{1} + \mu } \right)I\left( t \right), t \ge 0, \\ \frac{dV}{{dt}}  & =  \delta_{2} S\left( t \right) + \delta_{1} I\left( t \right) - \left( {\sigma + \mu } \right)V\left( t \right), t \ge 0, \\ \frac{dR}{{dt}}  & =  \gamma I\left( t \right) + \sigma V\left( t \right) - \mu R\left( t \right), t \ge 0 . \\ \end{aligned} $$

The total dynamics of the system (1) is obtained by adding the four equations as follows:

2 $$ \frac{dS}{{dt}} + \frac{dI}{{dt}} + \frac{dV}{{dt}} + \frac{dR}{{dt}} = \Lambda - \mu N, $$

where $$S + I + V + R = N$$.

We have

3 $$ \frac{dN}{{dt}} = \Lambda - \mu N $$

and hence,

4 $$ N\left( t \right) \le \frac{\Lambda }{\mu }, \;{\text{whenever}}\;t \to \infty . $$

The feasible region of the system (1) is defined in the following way

5 $$ \Omega = \left\{ {S\left( t \right),I\left( t \right),V\left( t \right),R\left( t \right) \in R_{ + }^{4} : N\left( t \right) \le \frac{\Lambda }{\mu }} \right\} $$

### Properties

#### Theorem 1

(Positivity) For any initial data $\left( {S\left( 0 \right), I\left( 0 \right), V\left( 0 \right), R\left( 0 \right)} \right) \in R_{ + }^{4}$, then the solution $\left( {S\left( t \right),I\left( t \right),V\left( t \right),R\left( t \right)} \right)$ for the system (1) is positive invariant set in ${R}_{+}^{4}.$

#### Proof

Let us start from the class $I\left( t \right)$,

$$ I\left( t \right) \ge I\left( 0 \right)e^{{ - \left( {\gamma + \delta_{1} + \mu } \right)t }} \ge 0,\forall t \ge 0. $$

For the function $V\left( t \right),$ the following inequalities hold:

$$ V\left( t \right) \ge V\left( 0 \right)e^{{ - \left( {\sigma + \mu } \right)t }} \ge 0,\forall t \ge 0, $$

and

$$ R\left( t \right) \ge R\left( 0 \right)e^{ - \mu t } \ge 0,\forall t \ge 0. $$

We shall define the norm

$$ \lambda_{\infty } = \sup_{{t \in D_{\lambda } }} \left| {\lambda \left( t \right)} \right|, $$

where $D_{\lambda }$ is the domain of $\lambda$. Using the above norm, the inequalities for the function $S\left( t \right)$ are defined

$$ \frac{dS}{{dt}} = \mu N - \frac{\beta SI}{{1 + \alpha I}} - (\delta_{2} + \mu )S,\forall t \ge 0, $$

$$ \frac{dS}{{dt}} \ge - \left( {\delta_{2} + \mu + \frac{\beta \left| I \right|}{{1 + \alpha \left| I \right|}}} \right)S,\forall t \ge 0, $$

$$ \frac{dS}{{dt}} \ge - \left( {\delta_{2} + \mu + \frac{{\beta \sup_{{t \in D_{\lambda } }} \left| I \right|}}{{1 + \alpha \sup_{{t \in D_{\lambda } }} \left| I \right|}}} \right)S,\forall t \ge 0, $$

$$ \frac{dS}{{dt}} \ge - \left( {\delta_{2} + \mu + \frac{{\beta I_{\infty } }}{{1 + \alpha I_{\infty } }}} \right)S,\forall t \ge 0, $$

$$ S\left( t \right) \ge S\left( 0 \right)e^{{ - \left( {\delta_{2} + \mu + \frac{{\beta I_{\infty } }}{{1 + \alpha I_{\infty } }}} \right)t }} \ge 0, $$

as desired.

#### Theorem 2

(Boundedness) For any time t, the system (1) is bounded and lies in the feasible region ${\Omega }$, if $\mathop {\lim }\limits_{t \to \infty } Sup N\left( t \right) \le \frac{\Lambda }{\mu }.$

#### Proof

By letting the population function $N\left( t \right) = S\left( t \right) + I\left( t \right) + V\left( t \right) + R\left( t \right)$,

$$ \frac{dN}{{dt}} = \frac{dS}{{dt}} + \frac{dI}{{dt}} + \frac{dV}{{dt}} + \frac{dR}{{dt}},\frac{dN}{{dt}} = \Lambda - \mu N = 0, $$

$$ N\left( t \right) = N\left( 0 \right)e^{ - \mu t} + \frac{\Lambda }{\mu },\mathop {\lim }\limits_{t \to \infty } Sup N\left( t \right) \le \frac{\Lambda }{\mu }, $$

as desired.

We shall define the norm $\lambda_{\infty } = \sup_{{t \in D_{\lambda } }} \left| {\lambda \left( t \right)} \right|$, and we consider the Banach space [27]. We present here the existence and uniqueness of the solution piece wisely. To obtain such results, we need to verify growth and Lipschitz condition properties. Let us consider the four positive constants $M_{1} , M_{2} , M_{3}$, and $M_{4} < \infty$ such that $S_{\infty } < M_{1}$, $I_{\infty } < M_{2} , V_{\infty } < M_{3} ,$ and $R_{\infty } < M_{4}$. We have

6 $$ \left\{ {\begin{array}{*{20}c} {\begin{array}{*{20}c} {S^{\prime } = f_{1} \left( {S,I,V,R,t} \right)} \\ {I^{\prime } = f_{2} \left( {S,I,V,R,t} \right)} \\ \end{array} } \\ {V^{\prime } = f_{3} \left( {S,I,V,R,t} \right)} \\ {R^{\prime } = f_{4} \left( {S,I,V,R,t} \right)} \\ \end{array} } \right.,\,\;\forall t \ge 0 $$

$\forall i = 1,2,3,4$. We first verify that

7 $$ \left| {f_{i} \left( {S,t} \right)} \right|^{2} < k_{i} \left( {\left| {S_{i} } \right|^{2} + 1} \right), $$

8 $$ \left| {f_{i} \left( {S^{1} ,t} \right) - f_{i} \left( {S^{2} ,t} \right)} \right|^{2} < \overline{{k_{i} }} \left| {S^{1} - S^{2} } \right|^{2} . $$

For proof, we consider the function $f_{1} \left( {S,I,V,R,t} \right)$, and the following estimations hold

9 $$ \left| {f_{1} \left( {S,I,V,R,t} \right)} \right|^{2} = \left| {\Lambda - \frac{\beta SI}{{1 + \alpha I}} - \left( {\delta_{2} + \mu } \right)S} \right|^{2} , $$

$$ \left| {f_{1} \left( {S,I,V,R,t} \right)} \right|^{2} \le 4\left| \Lambda \right|^{2} + 4\left| {\frac{\beta SI}{{1 + \alpha I}}} \right|^{2} + 4\left| {\left( {\delta_{2} + \mu } \right)S} \right|^{2} , $$

$$ \left| {f_{1} \left( {S,I,V,R,t} \right)} \right|^{2} \le 4\left( {\left| \Lambda \right|^{2} + \sup_{{t \in D_{\lambda } }} \left| {\frac{\beta SI}{{1 + \alpha I}}} \right|^{2} + \sup_{{t \in D_{\lambda } }} \left| {\left( {\delta_{2} + \mu } \right)S} \right|^{2} } \right), $$

$$ \left| {f_{1} \left( {S,I,V,R,t} \right)} \right|^{2} \le 4\left( {\left| \Lambda \right|^{2} + \left| \beta \right|\frac{SI}{{1 + \alpha I}}_{\infty } + \left| {\left( {\delta_{2} + \mu } \right)} \right|^{2} S_{\infty } } \right), $$

$$ \left| {f_{1} \left( {S,I,V,R,t} \right)} \right|^{2} \le 4\left( {\left| \Lambda \right|^{2} + \left| {\left( {\delta_{2} + \mu } \right)} \right|^{2} S_{\infty } } \right)\left( {1 + \frac{{\left| \beta \right|\frac{SI}{{1 + \alpha I}}_{\infty } }}{{\left( {\left| \Lambda \right|^{2} + \left| {\left( {\delta_{2} + \mu } \right)} \right|^{2} S_{\infty } } \right)}}} \right). $$

The condition $\frac{{\left| \beta \right|\frac{SI}{{1 + \alpha I}}_{\infty } }}{{\left( {\left| \Lambda \right|^{2} + \left| {\left( {\delta_{2} + \mu } \right)} \right|^{2} S_{\infty } } \right)}} < 1$, implies

10 $$ \left| {f_{1} \left( {S,I,V,R,t} \right)} \right|^{2} < k_{1} \left( {1 + \left| S \right|^{2} } \right). $$

By using the same methodology, we get

11 $$ \left| {f_{2} \left( {S,I,V,R,t} \right)} \right|^{2} = \left| {\frac{\beta SI}{{1 + \alpha I}} - \left( {\gamma + \delta_{1} + \mu } \right)I} \right|^{2} , $$

$$ \left| {f_{2} \left( {S,I,V,R,t} \right)} \right|^{2} \le 3\left| {\frac{\beta SI}{{1 + \alpha I}}} \right|^{2} + 3\left| {\left( {\gamma + \delta_{1} + \mu } \right)I} \right|^{2} , $$

$$ \left| {f_{2} \left( {S,I,V,R,t} \right)} \right|^{2} \le 3\left( {\sup_{{t \in D_{\lambda } }} \left| {\frac{\beta SI}{{1 + \alpha I}}} \right|^{2} + \sup_{{t \in D_{\lambda } }} \left| {\left( {\gamma + \delta_{1} + \mu } \right)I} \right|^{2} } \right), $$

$$ \left| {f_{2} \left( {S,I,V,R,t} \right)} \right|^{2} \le 3\left( {\left| \beta \right|\frac{SI}{{1 + \alpha I}}_{\infty } + \left| {\left( {\gamma + \delta_{1} + \mu } \right)} \right|^{2} I_{\infty } } \right), $$

$$ \left| {f_{2} \left( {S,I,V,R,t} \right)} \right|^{2} \le 3\left( {\left| {\left( {\gamma + \delta_{1} + \mu } \right)} \right|^{2} I_{\infty } } \right)\left( {1 + { }\frac{{\left| \beta \right|\frac{SI}{{1 + \alpha I}}_{\infty } }}{{\left( {\left| {\left( {\gamma + \delta_{1} + \mu } \right)} \right|^{2} I_{\infty } } \right)}}} \right) $$

Under the condition that $\frac{{\left| \beta \right|\frac{SI}{{1 + \alpha I}}_{\infty } }}{{\left( {\left| {\left( {\gamma + \delta_{1} + \mu } \right)} \right|^{2} I_{\infty } } \right)}} < 1$, we estimate

12 $$ \left| {f_{2} \left( {S,I,V,R,t} \right)} \right|^{2} < k_{2} \left( {1 + \left| I \right|^{2} } \right) $$

For the function $f_{3} ,$. We have

13 $$ \left| {f_{3} \left( {S,I,V,R,t} \right)} \right|^{2} = \left| {\delta_{2} S + \delta_{1} I - \left( {\sigma + \mu } \right)V} \right|^{2} , $$

$$ \left| {f_{3} \left( {S,I,V,R,t} \right)} \right|^{2} \le 2\left| {\delta_{2} S + \delta_{1} I} \right|^{2} + 2\left| {\left( {\sigma + \mu } \right)V} \right|^{2} , $$

$$ \left| {f_{3} \left( {S,I,V,R,t} \right)} \right|^{2} \le 2\left( {\sup_{{t \in D_{\lambda } }} \left| {\delta_{2} S + \delta_{1} I} \right|^{2} + \sup_{{t \in D_{\lambda } }} \left| {\left( {\sigma + \mu } \right)V} \right|^{2} } \right), $$

$$ \left| {f_{3} \left( {S,I,V,R,t} \right)} \right|^{2} \le 2\left( {\left| {\delta_{2} } \right|^{2} S_{\infty } + \left| {\delta_{1} } \right|^{2} I_{\infty } + \left| {\left( {\sigma + \mu } \right)} \right|^{2} V_{\infty } } \right), $$

$$ \left| {f_{3} \left( {S,I,V,R,t} \right)} \right|^{2} \le 2\left( {\left| {\left( {\sigma + \mu } \right)} \right|^{2} V_{\infty } } \right)\left( {1 + \frac{{\left| {\delta_{2} } \right|^{2} S_{\infty } + \left| {\delta_{1} } \right|^{2} I_{\infty } }}{{\left( {\left| {\left( {\sigma + \mu } \right)} \right|^{2} V_{\infty } } \right)}}} \right). $$

The condition $\frac{{\left| {\delta_{2} } \right|^{2} S_{\infty } + \left| {\delta_{1} } \right|^{2} I_{\infty } }}{{\left( {\left| {\left( {\sigma + \mu } \right)} \right|^{2} V_{\infty } } \right)}} < 1$ yields

14 $$ \left| {f_{3} \left( {S,I,V,R,t} \right)} \right|^{2} < k_{3} \left( {1 + \left| V \right|^{2} } \right). $$

For the function $f_{4} ,$ we have

15 $$ \left| {f_{4} \left( {S,I,V,R,t} \right)} \right|^{2} = \left| {\gamma I + \delta V - \mu R} \right|^{2} , $$

$$ \left| {f_{4} \left( {S,I,V,R,t} \right)} \right|^{2} \le \left| {\gamma I + \delta V} \right|^{2} + \left| {\mu R} \right|^{2} , $$

$$ \left| {f_{4} \left( {S,I,V,R,t} \right)} \right|^{2} \le \left( {\sup_{{t \in D_{\lambda } }} \left| {\gamma I + \delta V} \right|^{2} + \sup_{{t \in D_{\lambda } }} \left| {\mu R} \right|^{2} } \right), $$

$$ \left| {f_{4} \left( {S,I,V,R,t} \right)} \right|^{2} \le \left( {\left| \gamma \right|^{2} I_{\infty } + \left| \delta \right|^{2} V_{\infty } + \left| \mu \right|^{2} R_{\infty } } \right), $$

$$ \left| {f_{4} \left( {S,I,V,R,t} \right)} \right|^{2} \le \left| \mu \right|^{2} R_{\infty } \left( {1 + \frac{{\left| \gamma \right|^{2} I_{\infty } + \left| \delta \right|^{2} V_{\infty } }}{{\left| \mu \right|^{2} R_{\infty } }}} \right). $$

The condition $\frac{{\left| \gamma \right|^{2} I_{\infty } + \left| \delta \right|^{2} V_{\infty } }}{{\left| \mu \right|^{2} R_{\infty } }} < 1$ implies that

16 $$ \left| {f_{4} \left( {S,I,V,R,t} \right)} \right|^{2} < k_{4} \left( {1 + \left| R \right|^{2} } \right) $$

Therefore, the condition of linear growth is verified if

$$\max \left\{ {\frac{{\left| \beta \right|\frac{SI}{{1 + \alpha I}}_{\infty } }}{{\left( {\left| \Lambda \right|^{2} + \left| {\left( {\delta_{2} + \mu } \right)} \right|^{2} S_{\infty } } \right)}},\frac{{\left| \beta \right|\frac{SI}{{1 + \alpha I}}_{\infty } }}{{\left( {\left| {\left( {\gamma + \delta_{1} + \mu } \right)} \right|^{2} I_{\infty } } \right)}},\frac{{\left| {\delta_{2} } \right|^{2} S_{\infty } + \left| {\delta_{1} } \right|^{2} I_{\infty } }}{{\left( {\left| {\left( {\sigma + \mu } \right)} \right|^{2} V_{\infty } } \right)}}, \frac{{\left| \gamma \right|^{2} I_{\infty } + \left| \delta \right|^{2} V_{\infty } }}{{\left| \mu \right|^{2} R_{\infty } }} } \right\} < 1,$$

as desired.

### Equilibria

We determine the equilibria of the system (1) by assuming that the state variables are constant and by putting the right side equal to zero. Equation (1) admits two types of equilibria as follows:

(i) coronavirus-free equilibrium = $C_{1} = \left( {S^{1} ,I^{1} ,V^{1} ,R^{1} } \right) = \left( {\frac{\Lambda }{{\delta_{2} + \mu }},0,0,0} \right)$,
(ii) corona existing equilibrium = $C_{2} = \left( {S^{*} ,I^{*} ,V^{*} ,R^{*} } \right)$,

where: $$S^{*} = \left( {\frac{{\gamma + \delta_{1} + \mu }}{\beta }} \right)\left( {1 + \alpha I^{*} } \right)$$, $$B_{1} I^{{{*}^{2} }} + B_{2} I^{*} + B_{3} = 0,$$ $$B_{1} = \left( {\gamma + \delta_{1} + \mu } \right) + \alpha \left( {\delta_{2} + u} \right)\left( {\gamma + \delta_{1} + \mu } \right),$$ $$B_{2} = \left( {\delta_{2} + u} \right)\left( {\frac{{\gamma + \delta_{1} + \mu }}{\beta }} \right)\alpha + \left( {\delta_{2} + \mu } \right)\alpha \left( {\frac{{\gamma + \delta_{1} + \mu }}{\beta }} \right) + \beta \left( {\frac{{\gamma + \delta_{1} + \mu }}{\beta }} \right) - {\Lambda }\alpha$$, $$B_{3} = { }\left( {\delta_{2} + u} \right)\left( {\frac{{\gamma + \delta_{1} + \mu }}{\beta }} \right) - {\Lambda }$$, $$I^{*} = \frac{{ - B_{2} + \sqrt {B_{2}^{2} - 4B_{1} B_{3} } }}{{2B_{1} }}$$, $$V^{*} = \frac{{\delta_{2} S^{*} + \delta_{1} I^{*} }}{\sigma + \mu }$$, $$R^{*} = \frac{{\gamma I^{*} + \sigma V^{*} }}{\mu }$$.

### Reproduction Number

We determine the reproduction number of the system (1) by using the well-known results like the next-generation matrix method after substituting the value of coronavirus-free equilibrium. We get

$$ \left[ {\begin{array}{*{20}c} {I^{\prime}} \\ {V^{\prime}} \\ {R^{\prime}} \\ \end{array} } \right] = \left[ {\begin{array}{*{20}c} {\frac{\beta \Lambda }{{\left( {\delta_{2} + \mu } \right)}}} & 0 & 0 \\ 0 & 0 & 0 \\ 0 & 0 & 0 \\ \end{array} } \right]\left[ {\begin{array}{*{20}c} I \\ V \\ R \\ \end{array} } \right] - \left[ {\begin{array}{*{20}c} {\left( {\gamma + \delta_{1} + \mu } \right)} & 0 & 0 \\ { - \delta_{1} \left( {\sigma + \mu } \right)} & 0 & 0 \\ { - r} & { - \sigma } & \mu \\ \end{array} } \right]\left[ {\begin{array}{*{20}c} I \\ V \\ R \\ \end{array} } \right], $$

where: $$A = \left[ {\begin{array}{*{20}c} {\frac{\beta \Lambda }{{\left( {\delta_{2} + \mu } \right)}}} & 0 & 0 \\ 0 & 0 & 0 \\ 0 & 0 & 0 \\ \end{array} } \right],B = \left[ {\begin{array}{*{20}c} {\left( {\gamma + \delta_{1} + \mu } \right)} & 0 & 0 \\ { - \delta_{1} \left( {\sigma + \mu } \right)} & 0 & 0 \\ { - r} & { - \sigma } & \mu \\ \end{array} } \right],$$

$$ AB^{ - 1} = \left[ {\begin{array}{*{20}c} {\frac{{\beta {\Lambda }}}{{\left( {\delta_{2} + \mu } \right)\left( {\gamma + \delta_{1} + \mu } \right)}}} & 0 & 0 \\ 0 & 0 & 0 \\ 0 & 0 & 0 \\ \end{array} } \right] $$

The spectral radius of $\rho \left( {AB^{ - 1} } \right)$ is denoted by $R_{0} = \frac{{\beta {\Lambda }}}{{\left( {\delta_{2} + \mu } \right)\left( {\gamma + \delta_{1} + \mu } \right)}}$.

### Strength number

The extension of the reproduction number is called the strength number. No doubt, the reproduction number has great significance in the field of epidemiology regarding the spread and extinction of disease. The reason behind different techniques used by the epidemiologist is to obtain such numbers (i.e., violation of uniqueness of reproduction number and many more). The primary outcome of this method relies on the prediction of the waves of the spread of disease. The critical thing is that the next-generation matrix method is used to evaluate this number by assuming the coronavirus-free equilibrium into the system (1) by taking the second derivative of infectious classes. Consequently, the transmission and transition matrices are represented by $F$ and $G$ [25], where:

$$ F = \left[ {\begin{array}{*{20}c} {\frac{\beta \Lambda }{{\left( {\delta_{2} + \mu } \right)}}} & 0 & 0 \\ 0 & 0 & 0 \\ 0 & 0 & 0 \\ \end{array} } \right],G^{ - 1} = \left[ {\begin{array}{*{20}c} {\frac{1}{{\left( {\gamma + \delta_{1} + \mu } \right)}}} & 0 & 0 \\ {\frac{{\delta_{1} }}{{\left( {\gamma + \delta_{1} + \mu } \right)\left( {\sigma + \mu } \right)}}} & {\frac{1}{{\left( {\sigma + \mu } \right)}}} & 0 \\ {\frac{{\delta_{1} \sigma + \gamma \left( {\sigma + \mu } \right)}}{{\mu \left( {\gamma + \delta_{1} + \mu } \right)\left( {\sigma + \mu } \right)}}} & {\frac{\sigma }{{\mu \left( {\sigma + \mu } \right)}}} & {\frac{1}{\mu }} \\ \end{array} } \right], $$

$$ FG^{ - 1} = \left[ {\begin{array}{*{20}c} { - \frac{{\beta {\Lambda }}}{{\left( {\delta_{2} + \mu } \right)}}} & 0 & 0 \\ 0 & 0 & 0 \\ 0 & 0 & 0 \\ \end{array} } \right]. $$

Here, $A_{0} = - \frac{{\beta {\Lambda }}}{{\left( {\delta_{2} + \mu } \right)}} < 0$ is called the strength number of the system (1). The remarkable conclusion includes the analysis of local maximum, local minimum, and inflection points. Thus, having a negative strength number is an indication that the system (1) will have a single magnitude, either a maximum with two infection points indicating a single wave or a rapid decrease from the coronavirus-free equilibrium. Thus, the infection will rise after a minimum point with the renewal process and then be stabilized or stopped later on, as desired.

### Stability analysis

In this section, we test the local and global stability of the system (1), considering the two defined equilibria.

#### Theorem 3

(Local stability at $$C_{1} )$$ The system (1) at $$C_{1} = \left( {S^{1} ,I^{1} , V^{1} ,R^{1} } \right) = \left( {\frac{\Lambda }{{\delta_{2} + \mu }},0,0,0} \right)$$ is locally asymptotically stable if $$R_{0} < 1$$. Otherwise, unstable when $$R_{0} > 1$$.

#### Proof

The Jacobian matrix obtained from the system (1) is as follows.

$$ J\left( {S,I,V,R} \right) = \left[ {\begin{array}{*{20}c} { - \frac{\beta I}{{1 + \alpha I}} - \delta_{2} - \mu } & { - \frac{\beta S}{{\left( {1 + \alpha I} \right)^{2} }}} & 0 & 0 \\ {\frac{\beta I}{{1 + \alpha I}}} & {\frac{\beta S}{{\left( {1 + \alpha I} \right)^{2} }} - \gamma - \delta_{1} - \mu } & 0 & 0 \\ {\delta_{2} } & {\delta_{1} } & { - \sigma - \mu } & 0 \\ 0 & \gamma & \sigma & { - \mu } \\ \end{array} } \right]. $$

The Jacobian matrix at $C_{1}$ is as follows

$$ J\left( {\frac{\Lambda }{{\delta_{2} + \mu }},0,0,0} \right) = \left[ {\begin{array}{*{20}c} { - \delta_{2} - \mu } & { - \frac{\beta \Lambda }{{\delta_{2} + \mu }}} & 0 & 0 \\ {\frac{\beta I}{{1 + \alpha I}}} & {\frac{\beta \Lambda }{{\delta_{2} + \mu }} - \gamma - \delta_{1} - \mu } & 0 & 0 \\ {\delta_{2} } & {\delta_{1} } & { - \sigma - \mu } & 0 \\ 0 & \gamma & \sigma & { - \mu } \\ \end{array} } \right] $$

Consider $\left| {J - \lambda I} \right| = 0$, and hence

$$ \left| {\begin{array}{*{20}c} { - \delta_{2} - \mu - \lambda } & { - \frac{\beta \Lambda }{{\delta_{2} + \mu }}} & 0 & 0 \\ 0 & {\frac{\beta \Lambda }{{\delta_{2} + \mu }} - \gamma - \delta_{1} - \mu - \lambda } & 0 & 0 \\ {\delta_{2} } & {\delta_{1} } & { - \sigma - \mu - \lambda } & 0 \\ 0 & \gamma & \sigma & { - \mu - \lambda } \\ \end{array} } \right| = 0, $$

$$\lambda_{1} = - \mu < 0$$, $$\lambda_{2} = - \left( {\sigma + \mu } \right) < 0$$, $$\lambda_{3} = - (\delta_{2} + \mu ) < 0$$, and.

$$\lambda_{4} = \frac{\beta \Lambda }{{\delta_{2} + \mu }} - \gamma - \delta_{1} - \mu < 0$$, $$\frac{\beta \Lambda }{{\left( {\delta_{2} + \mu } \right)\left( {\gamma + \delta_{1} + \mu } \right)}} < 1, R_{0} < 1.$$

It is seen that $C_{1}$ is locally asymptotically stable (LAS), $R_{0} < 1.$

#### Theorem 4

(Local stability at $$C_{2} )$$ The system (1) at $$ C_{2} = \left( {S^{*} ,I^{*} ,V^{*} ,R^{*} } \right)$$ is locally asymptotically stable if $$R_{0} > 1$$.

#### Proof

The Jacobian matrix at $C_{2}$ of the system (1) is as follows.

$$J\left( {S^{*} ,I^{*} ,V^{*} ,R^{*} } \right) = \left[ {\begin{array}{*{20}c} { - \frac{{\beta I^{*} }}{{1 + \alpha I^{*} }} - \delta_{2} - \mu } & { - \frac{{\beta S^{*} }}{{\left( {1 + \alpha I^{*} } \right)^{2} }}} & 0 & 0 \\ {\frac{{\beta I^{*} }}{{1 + \alpha I^{*} }}} & {\frac{{\beta S^{*} }}{{\left( {1 + \alpha I^{*} } \right)^{2} }} - \gamma - \delta_{1} - \mu } & 0 & 0 \\ {\delta_{2} } & {\delta_{1} } & { - \sigma - \mu } & 0 \\ 0 & \gamma & \sigma & { - \mu } \\ \end{array} } \right]$$.

Consider $\left| {J - \lambda I} \right| = 0$, which implies

$$ \left| {\begin{array}{*{20}c} { - \frac{{\beta I^{*} }}{{1 + \alpha I^{*} }} - \delta_{2} - \mu - \lambda } & { - \frac{{\beta S^{*} }}{{\left( {1 + \alpha I^{*} } \right)^{2} }}} & 0 & 0 \\ {\frac{{\beta I^{*} }}{{1 + \alpha I^{*} }}} & {\frac{{\beta S^{*} }}{{\left( {1 + \alpha I^{*} } \right)^{2} }} - \gamma - \delta_{1} - \mu - \lambda } & 0 & 0 \\ {\delta_{2} } & {\delta_{1} } & { - \sigma - \mu - \lambda } & 0 \\ 0 & \gamma & \sigma & { - \mu - \lambda } \\ \end{array} } \right| = 0, $$

$$\lambda_{1} = - \mu < 0$$, $$\lambda_{2} = - \left( {\sigma + \mu } \right) < 0,$$

$$ \lambda^{2} + \left( {\frac{{\beta I^{*} }}{{1 + \alpha I^{*} }} - \frac{{\beta S^{*} }}{{\left( {1 + \alpha I^{*} } \right)^{2} }} + \delta_{1} + \delta_{2} + \gamma + 2\mu } \right)\lambda + \left( {\gamma + \delta_{1} + \mu } \right)\left( {\frac{{\beta I^{*} }}{{1 + \alpha I^{*} }} + \delta_{2} + \mu } \right) - \frac{{\left( {\delta_{2} + \mu } \right)\beta S^{*} }}{{\left( {1 + \alpha I^{*} } \right)^{2} }} = 0, $$

$$ \lambda^{2} + A_{1} \lambda + A_{0} = 0, $$

where:

$$A_{1} = \frac{{\beta I^{*} }}{{1 + \alpha I^{*} }} - \frac{{\beta S^{*} }}{{\left( {1 + \alpha I^{*} } \right)^{2} }} + \delta_{1} + \delta_{2} + \gamma + 2\mu ,$$,

$$ A_{0} = \left( {\gamma + \delta_{1} + \mu } \right)\left( {\frac{{\beta I^{*} }}{{1 + \alpha I^{*} }} + \delta_{2} + \mu } \right) - \frac{{\left( {\delta_{2} + \mu } \right)\beta S^{*} }}{{\left( {1 + \alpha I^{*} } \right)^{2} }}. $$

Since $A_{0} , A_{1}$ both are positive when $R_{0} > 1,$ by the Routh–Hurwitz criterion for the second order, the $C_{2}$ is locally asymptotically stable.

#### Theorem 5

(Global stability at $$C_{1} )$$ The system (1) at $$C_{1} = \left( {S^{1} ,I^{1} , V^{1} ,R^{1} } \right) = \left( {\frac{\Lambda }{{\delta_{2} + \mu }},0,0,0} \right)$$ is globally asymptotically stable if $$R_{0} < 1$$.

#### Proof

By letting the Lyapunov function $U: {\Omega } \to R$ defined as.

$$ U\left( I \right) = \ln \left( {\frac{I}{{I_{0} }}} \right), $$

$$ \frac{dU\left( I \right)}{{dt}} = \frac{{I^{\prime } }}{I} \times \frac{1}{{I^{\prime } }}\frac{dI}{{dt}}, $$

$$ \frac{dU\left( I \right)}{{dt}} = \frac{1}{I}\left[ {\frac{\beta SI}{{1 + \alpha I}} - \left( {\gamma + \delta_{1} + \mu } \right)I} \right], $$

$$ \frac{dU\left( I \right)}{{dt}} = \frac{\beta S}{{1 + \alpha I}} - \left( {\gamma + \delta_{1} + \mu } \right), $$

$$ \frac{dU}{{dt}} = \left( {\gamma + \delta_{1} + \mu } \right)\left[ {\frac{\beta SI}{{\left( {1 + \alpha I} \right)\left( {\gamma + \delta_{1} + \mu } \right)}} - 1} \right], $$

$$ \frac{dU}{{dt}} \le \left( {\gamma + \delta_{1} + \mu } \right)\left[ {\frac{\beta \Lambda }{{\left( {\delta_{2} + \mu } \right)\left( {\gamma + \delta_{1} + \mu } \right)}} - 1} \right], $$

$$\frac{dU}{{dt}} \le \left( {\gamma + \delta_{1} + \mu } \right)\left( {R_{0} - 1} \right)$$, $$\frac{dU}{{dt}} \le 0$$, if $$R_{0} < 1.$$

Hence, the system is globally asymptotically stable at $C_{1}$.

#### Theorem 6

(Global stability at $$C_{2} )$$ The system (1) at $$C_{2} = \left( {S^{*} ,I^{*} , V^{*} ,R^{*} } \right)$$ is globally asymptotically stable if $$R_{0} > 1$$.

#### Proof

By letting the Lyapunov function $Z: {\Omega } \to R$ defined as.

$$ \begin{aligned} Z  & =  K_{1} \left( {S - S^{*} - S^{*} \frac{\log S}{{S^{*} }}} \right) + K_{2} \left( {I - I^{*} - I^{*} \frac{\log I}{{I^{*} }}} \right) \\ & + K_{3} \left( {V - V^{*} - V^{*} \frac{\log V}{{V^{*} }}} \right) + K_{4} \left( {R - R^{*} - R^{*} \frac{\log R}{{R^{*} }}} \right) , \\ \end{aligned} $$

where $K_{i} \left( {i = 1,2,3,4} \right)$ are positive constants to be chosen later. We have

$$ \begin{aligned} \frac{dZ}{{dt}}  & =  K_{1} \left( {\frac{{s - s^{*} }}{s}} \right)\left( {\Lambda - \frac{\beta SI}{{1 + \alpha I}} - \delta_{2} S - \mu S} \right) + K_{2} \left( {\frac{{I - I^{*} }}{I}} \right)\left( {\frac{\beta SI}{{1 + \alpha I}} - I\gamma - \delta_{1} I - \mu I} \right) \\ & + K_{3} \left( {\frac{{V - V^{*} }}{V}} \right)\left( {\delta_{2} S + \delta_{1} I - \sigma V - \mu V} \right) + K_{4} \left( {\frac{{R - R^{*} }}{R}} \right)\left( {\gamma I + \sigma V - \mu R} \right), \\ \end{aligned} $$

$$ \begin{aligned} \frac{dZ}{{dt}}  & =  K_{1} \left( {s - s^{*} } \right)\left( {\frac{\Lambda }{s} - \frac{\beta I}{{1 + \alpha I}} - \delta_{2} - \mu } \right) + K_{2} \left( {I - I^{*} } \right)\left( {\frac{\beta S}{{1 + \alpha I}} - \gamma - \delta_{1} - \mu } \right) \\ & + K_{3} \left( {V - V^{*} } \right)\left( {\frac{{\delta_{2} S}}{V} + \frac{{\delta_{1} I}}{V} - \sigma - \mu } \right) + K_{4} \left( {R - R^{*} } \right)\left( {\frac{\gamma I}{R} + \frac{\sigma V}{R} - \mu } \right). \\ \end{aligned} $$

If we choose $K_{i} = 1,\left( {i = 1,2,3,4} \right)$, then.

$\frac{dZ}{{dt}} = \frac{{\Lambda \left( {S - S^{*} } \right)^{2} }}{{ss^{*} }} - \frac{{\beta SI\left( {I - I^{*} } \right)^{2} }}{{I^{*} \left( {1 + \alpha I} \right)}} - \frac{{\delta_{2} S\left( {V - V^{*} } \right)^{2} }}{{VV^{*} }} - \frac{{\delta_{2} I\left( {V - V^{*} } \right)^{2} }}{{VV^{*} }} - \frac{{\gamma I\left( {R - R^{*} } \right)^{2} }}{{RR^{*} }} - \frac{{\sigma V\left( {R - R^{*} } \right)^{2} }}{{RR^{*} }},$ $\frac{dZ}{{dt}} \le 0,$ for $R_{0} > 1$, and $\frac{dZ}{{dt}} = 0$ only if $S = S^{*} ,I = I^{*} ,V = V^{*} ,R = R^{*}$.

Hence, by Lasalle’s invariance principle, $C_{2}$ is globally asymptotically stable (GAS) in ${\Omega }$.

### Second derivative theory of Lyapunov stability

We present an analysis of the second derivative of the associated Lyapunov function of the system (1) to understand the variability of the process [26].

#### Theorem 7

(Global stability at $$C_{1} )$$ The system (1) at $$C_{1} = \left( {S^{1} ,I^{1} , V^{1} ,R^{1} } \right) = \left( {\frac{\Lambda }{{\delta_{2} + \mu }},0,0,0} \right)$$ is globally asymptotically stable if $$R_{0} < 1$$.

#### Proof

Consider,

$$ U^{\prime \prime } \left( I \right) = \frac{d}{dt}\left( {\ln \left( {\frac{I}{{I_{0} }}} \right)} \right), $$

$$ U^{\prime \prime } \left( I \right) = - \frac{1}{{I^{2} }}\left( {\frac{dI}{{dt}}} \right)^{2} + \frac{1}{I}\frac{{d^{2} I}}{{dt^{2} }}, $$

$$ U^{\prime \prime } \left( I \right) = - \frac{1}{{I^{2} }}\left( {\frac{\beta SI}{{1 + \alpha I}} - \left( {\gamma + \delta_{1} + \mu } \right)I} \right)^{2} + \frac{1}{I}\left( {\frac{\beta S}{{1 + \alpha I}} - \left( {\gamma + \delta_{1} + \mu } \right)\left( {\frac{\beta SI}{{1 + \alpha I}} - \left( {\gamma + \delta_{1} + \mu } \right)I} \right)} \right), $$

$$ U^{\prime \prime } \left( I \right) = - \left( {\frac{\beta S}{{1 + \alpha I}} - \left( {\gamma + \delta_{1} + \mu } \right)} \right)^{2} - \left( {\gamma + \delta_{1} + \mu } \right)^{2} \left( {1 - \frac{\beta \Lambda }{{\left( {\delta_{2} + \mu } \right)\left( {\gamma + \delta_{1} + \mu } \right)}}} \right), $$

$$ U^{\prime \prime } \left( I \right) = - \left( {\frac{\beta \Lambda }{{\left( {\delta_{2} + \mu } \right)}} - \left( {\gamma + \delta_{1} + \mu } \right)} \right)^{2} - \left( {\gamma + \delta_{1} + \mu } \right)^{2} \left( {1 - \frac{\beta \Lambda }{{\left( {\delta_{2} + \mu } \right)\left( {\gamma + \delta_{1} + \mu } \right)}}} \right), $$

$$ \frac{{d^{2} U}}{{dt^{2} }} \le - \left( {\frac{\beta \Lambda }{{\left( {\delta_{2} + \mu } \right)}} - \left( {\gamma + \delta_{1} + \mu } \right)} \right)^{2} - \left( {\gamma + \delta_{1} + \mu } \right)^{2} \left( {1 - R_{0} } \right), $$

$\frac{{d^{2} U}}{{dt^{2} }} \le 0$, if $R_{0} < 1$. Hence, at $C_{1}$, the system is globally asymptotically stable.

#### Theorem 8

(Global stability at $$C_{2} )$$ The system (1) at $$C_{2} = \left( {S^{*} ,I^{*} , V^{*} ,R^{*} } \right)$$ is globally asymptotically stable if $$R_{0} > 1$$.

#### Proof

Consider

$$Z^{\prime \prime } = \frac{d}{dt}\left[ {\left( {\frac{{S - S^{*} }}{S}} \right)\frac{dS}{{dt}} + \left( {\frac{{I - I^{*} }}{I}} \right)\frac{dI}{{dt}} + \left( {\frac{{V - V^{*} }}{V}} \right)\frac{dV}{{dt}} + \left( {\frac{{R - R^{*} }}{R}} \right)\frac{dR}{{dt}}} \right]$$,

$$ Z^{\prime \prime } = \frac{{S^{*} }}{{S^{2} }}\left( {\frac{dS}{{dt}}} \right)^{2} + \left( {\frac{{S - S^{*} }}{s}} \right)\frac{{d^{2} S}}{{dt^{2} }} + \frac{{I^{*} }}{{I^{2} }}\left( {\frac{dI}{{dt}}} \right)^{2} + \left( {\frac{{I - I^{*} }}{I}} \right)\frac{{d^{2} I}}{{dt^{2} }} + \frac{{V^{*} }}{{V^{2} }}\left( {\frac{dV}{{dt}}} \right)^{2} + \left( {\frac{{V - V^{*} }}{V}} \right)\frac{{d^{2} V}}{{dt^{2} }} + \frac{{R^{*} }}{{R^{2} }}\left( {\frac{dR}{{dt}}} \right)^{2} + \left( {\frac{{R - R^{*} }}{R}} \right)\frac{{d^{2} R}}{{dt^{2} }}, $$

Here, $$\frac{{d^{2} S}}{{dt^{2} }} = - \frac{\beta S}{{1 + \alpha I}} - \Lambda \left( {\delta_{2} + \mu } \right) + \frac{\beta SI}{{1 + \alpha I}}\left( {\delta_{2} + \mu } \right) + \left( {\delta_{2} + \mu } \right)^{2} S,$$,

$$ \frac{{d^{2} I}}{{dt^{2} }} = \frac{\beta S}{{1 + \alpha I}} - \left( {\gamma + \delta_{1} + \mu } \right)\frac{\beta SI}{{1 + \alpha I}} + \left( {\gamma + \delta_{1} + \mu } \right)^{2} I, $$

$$ \frac{{d^{2} V}}{{dt^{2} }} = \delta_{2} \left( {\Lambda - \frac{\beta SI}{{1 + \alpha I}} - \left( {\delta_{2} + \mu } \right)S} \right) + \delta_{1} \left( {\frac{\beta SI}{{1 + \alpha I}} - \left( {\gamma + \delta_{1} + \mu } \right)I} \right) - \left( {\delta_{2} \left( {\sigma + \mu } \right)S + \delta_{1} I\left( {\sigma + \mu } \right) - \left( {\sigma + \mu } \right)^{2} V} \right), $$

$$ \frac{{d^{2} R}}{{dt^{2} }} = \gamma \left( {\frac{\beta SI}{{1 + \alpha I}} - \left( {\gamma + \delta_{1} + \mu } \right)I} \right) + \delta \left( {\delta_{2} S + \delta_{1} I - \left( {\sigma + \mu } \right)V} \right) - \mu \left( {\gamma I + \delta V - \mu R} \right), $$

$$ \begin{aligned} \frac{{d^{2} Z}}{{dt^{2} }}  & =  \frac{{S^{*} }}{{S^{2} }}\left( {\Lambda - \frac{\beta SI}{{1 + \alpha I}} - \left( {\delta_{2} + \mu } \right)S} \right)^{2} + \left( {1 - \frac{{S^{*} }}{S}} \right)\left( { - \frac{\beta S}{{1 + \alpha I}} - \Lambda \left( {\delta_{2} + \mu } \right) + \frac{\beta SI}{{1 + \alpha I}}\left( {\delta_{2} + \mu } \right) + \left( {\delta_{2} + \mu } \right)^{2} S} \right) \\ & + \frac{{I^{*} }}{{I^{2} }}\left( {\frac{\beta SI}{{1 + \alpha I}} - \left( {\gamma + \delta_{1} + \mu } \right)I} \right)^{2} + \left( {1 - \frac{{I^{*} }}{I}} \right)\left( {\frac{\beta S}{{1 + \alpha I}} - \left( {\gamma + \delta_{1} + \mu } \right)\frac{\beta SI}{{1 + \alpha I}} + \left( {\gamma + \delta_{1} + \mu } \right)^{2} I} \right) \\ & + \frac{{V^{*} }}{{V^{2} }}\left( {\delta_{2} S + \delta_{1} I - \left( {\sigma + \mu } \right)V} \right)^{2} + \left( {1 - \frac{{V^{*} }}{V}} \right)\left( {\delta_{2} \left( {\Lambda - \frac{\beta SI}{{1 + \alpha I}} - \left( {\delta_{2} + \mu } \right)S} \right) + \delta_{1} \left( {\frac{\beta SI}{{1 + \alpha I}} - \left( {\gamma + \delta_{1} + \mu } \right)I} \right) - \left( {\delta_{2} \left( {\sigma + \mu } \right)S + \delta_{1} I\left( {\sigma + \mu } \right) - \left( {\sigma + \mu } \right)^{2} V} \right)} \right) \\ & + \frac{{R^{*} }}{{R^{2} }}\left( {\gamma I + \delta V - \mu R} \right)^{2} + \left( {1 - \frac{{R^{*} }}{R}} \right)\left( {\gamma \left( {\frac{\beta SI}{{1 + \alpha I}} - \left( {\gamma + \delta_{1} + \mu } \right)I} \right) + \delta \left( {\delta_{2} S + \delta_{1} I - \left( {\sigma + \mu } \right)V} \right) - \mu \left( {\gamma I + \delta V - \mu R} \right)} \right). \\ \end{aligned} $$

For simplicity, we choose $\frac{{d^{2} Z}}{{dt^{2} }} = \Psi_{1} - \Psi_{2}$, where:

$$ \begin{aligned} \Psi_{1}  & =  \frac{{S^{*} }}{{S^{2} }}\left( {\Lambda^{2} + \left( {\frac{\beta SI}{{1 + \alpha I}}} \right)^{2} + \left( {\delta_{2} + \mu } \right)^{2} S^{2} + \frac{{2\beta SI\left( {\delta_{2} + \mu } \right)S}}{1 + \alpha I}} \right) + \frac{\beta SI}{{1 + \alpha I}}\left( {\delta_{2} + \mu } \right) + \left( {\delta_{2} + \mu } \right)^{2} S \\ & + \frac{{S^{*} }}{S}\left( {\frac{\beta S}{{1 + \alpha I}}\Lambda \left( {\delta_{2} + \mu } \right)} \right) + \frac{{I^{*} }}{{I^{2} }}\left( {\left( {\frac{\beta SI}{{1 + \alpha I}}} \right)^{2} + \left( {\left( {\gamma + \delta_{1} + \mu } \right)I} \right)^{2} } \right) + \frac{\beta S}{{1 + \alpha I}} + \left( {\gamma + \delta_{1} + \mu } \right)^{2} I \\ & + \frac{{I^{*} }}{I}\left( {\left( {\gamma + \delta_{1} + \mu } \right)\frac{\beta SI}{{1 + \alpha I}}} \right) + \frac{{V^{*} }}{{V^{2} }}\left( {\left( {\delta_{2} S + \delta_{1} I} \right)^{2} + \left( {\left( {\sigma + \mu } \right)V} \right)^{2} } \right) + \left( {\delta_{2} \Lambda + \delta_{1} \frac{\beta SI}{{1 + \alpha I}} + \delta_{1} I\left( {\sigma + \mu } \right)} \right) \\ & + \frac{{V^{*} }}{V}\left( {\delta_{2} \left( {\frac{\beta SI}{{1 + \alpha I}} + \left( {\delta_{2} + \mu } \right)S} \right) + \delta_{1} \left( {\left( {\gamma + \delta_{1} + \mu } \right)I} \right) + \left( {\sigma + \mu } \right)^{2} V} \right) + \frac{{R^{*} }}{{R^{2} }}\left( {\left( {\gamma I + \delta V} \right)^{2} + \left( {\mu R} \right)^{2} } \right)\left( {\gamma I + \delta V - \mu R} \right)^{2} \\ & + \gamma \left( {\frac{\beta SI}{{1 + \alpha I}}} \right) + \delta \left( {\delta_{2} S + \delta_{1} I} \right) + \mu \left( {\gamma I + \delta V} \right) + \frac{{R^{*} }}{R}\left( {\left( {\gamma + \delta_{1} + \mu } \right)I + \left( {\sigma + \mu } \right)V + \mu \left( {\gamma I + \delta V} \right)} \right), \\ \end{aligned} $$

$$ \begin{aligned} \Psi_{2}  & =  \frac{2\Lambda \beta SI}{{1 + \alpha I}} + 2\Lambda \left( {\delta_{2} + \mu } \right)S + \frac{\beta S}{{1 + \alpha I}} + \Lambda \left( {\delta_{2} + \mu } \right) + \frac{{S^{*} }}{S}\left( {\frac{\beta SI}{{1 + \alpha I}}\left( {\delta_{2} + \mu } \right) + \left( {\delta_{2} + \mu } \right)^{2} S} \right) \\ & + \frac{{I^{*} }}{{I^{2} }}\left( {\frac{2\beta SI}{{1 + \alpha I}}} \right)\left( {\gamma + \delta_{1} + \mu } \right)I + \left( {\gamma + \delta_{1} + \mu } \right)\frac{\beta SI}{{1 + \alpha I}} + \frac{{I^{*} }}{I}\left( {\frac{\beta S}{{1 + \alpha I}} + \left( {\gamma + \delta_{1} + \mu } \right)^{2} I} \right) + 2\frac{{V^{*} }}{{V^{2} }}\left( {\delta_{2} S + \delta_{1} I} \right)\left( {\sigma + \mu } \right)V \\ & + \delta_{2} \left( {\frac{\beta SI}{{1 + \alpha I}} + \left( {\delta_{2} + \mu } \right)S} \right) + \delta_{1} \left( {\left( {\gamma + \delta_{1} + \mu } \right)I} \right) + \left( {\sigma + \mu } \right)^{2} V + \frac{{V^{*} }}{V}\left( {\delta_{2} \Lambda + \delta_{1} \frac{\beta SI}{{1 + \alpha I}} + \delta_{1} I\left( {\sigma + \mu } \right)} \right) \\ & + \frac{{2R^{*} }}{R}\mu \left( {\gamma I + \delta V} \right) + \left( {\gamma + \delta_{1} + \mu } \right)I + \left( {\sigma + \mu } \right)V + \mu \left( {\gamma I + \delta V} \right) + \frac{{R^{*} }}{R}\left( {\gamma \left( {\frac{\beta SI}{{1 + \alpha I}}} \right) + \delta \left( {\delta_{2} S + \delta_{1} I} \right) + \mu \left( {\gamma I + \delta V} \right)} \right). \\ \end{aligned} $$

It can be seen that

$$ \Psi_{1} > \Psi_{2} , \frac{{d^{2} Z}}{{dt^{2} }} > 0 ,\Psi_{1} < \Psi_{2} , \frac{{d^{2} Z}}{{dt^{2} }} < 0,\Psi_{1} = \Psi_{2} , \frac{{d^{2} Z}}{{dt^{2} }} = 0. $$

## Parameter estimations

For the realistic analysis and to predict the peak of the third strain of coronavirus in Pakistan, we need to use the real cases reported from August 01, 2021, till August 29, 2021, which is taken from the health ministry of Pakistan (www. Covid19. gov. pk) and presented in Table 1.

**Table 1:** Number of cases with different aspects reported in August 2021 [22]

Date	Confirmed cases	Active cases	Death	Recovered
1-Aug	4858	3457	40	1361
2-Aug	3582	2160	67	1355
3-Aug	4722	3222	46	1454
4-Aug	5661	1186	60	6787
5-Aug	4745	2583	67	2095
6-Aug	4720	155	95	4780
7-Aug	4455	2239	68	2148
8-Aug	4040	1222	53	2765
9-Aug	3884	1129	86	2669
10-Aug	4856	250	81	4525
11-Aug	4934	1456	102	3376
12-Aug	4619	603	79	3937
13-Aug	4786	370	73	4343
14-Aug	3711	603	67	3041
15-Aug	3669	1379	72	2218
16-Aug	3221	1165	95	4291
17-Aug	3974	786	66	3122
18-Aug	4373	1322	74	2977
19-Aug	3239	142	70	3027
20-Aug	3084	629	65	3648
21-Aug	3842	290	75	3477
22-Aug	3772	585	80	3107
23-Aug	4075	1127	91	2857
24-Aug	4199	158	126	3915
25-Aug	4467	954	100	3418
26-Aug	4056	726	95	3235
27-Aug	4191	223	120	3848
28-Aug	3909	397	69	3443
29-Aug	3800	186	66	3548

**Fig. 2:**
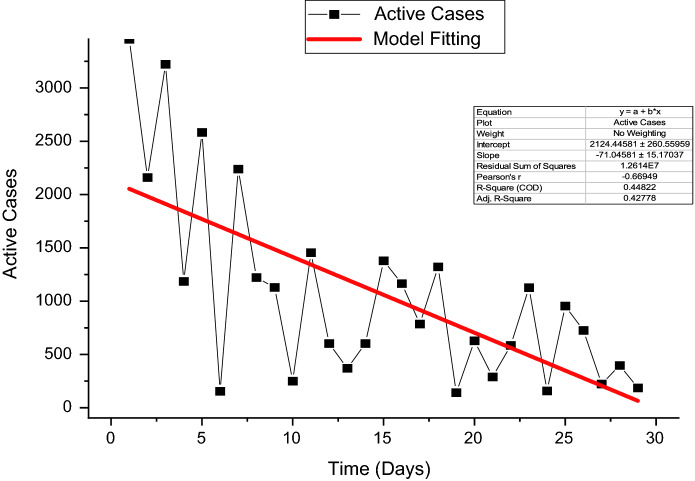
Reported cases from August 01, 2021, to August 29, 2021, versus model fit

Before we parametrize the system (1), we need to calculate the fundamental values of the parameters, such as the population's birth rate and death rate. According to the world meter info, the total population of Pakistan is $N\left(0\right)=$ 220,000,000 in 2021, while the birth rate of susceptible humans represented by $\Lambda$, and the death rate defined by $\mu$ are shown to be $\Lambda \simeq 8903$ per day and $\mu =1/(67.7\times 365)$, with the number ${\mu }_{av}=\frac{1}{67.7}$ as the average life span in Pakistan [24]. Furthermore, the values of the remaining parameters of the system (1) are presented in Table 2. Using the least-square curve fitting technique, we show the desired fitting in Fig. 2. The estimated value of the reproduction number is ${R}_{0}=1.3333$. Hence, the desired values of the transmission rates are instrumental in studying the system (1) graphically.

**Table 2:** Estimated values

Symbols	Value/per day	Source
${\varvec{\Lambda}}$	$\mu \times N(0)$	Estimated
${\varvec{\mu}}$	$\frac{1}{67.7\times 365}$	[23]
${\varvec{\gamma}}$	0.5000	Fitted
${\varvec{\beta}}$	0.4000	Fitted
$\boldsymbol{\alpha }$	0.00465	Fitted
${\varvec{\delta}}$	0.5038	Fitted
${{\varvec{\delta}}}_{1}$	0.1000	Fitted
${{\varvec{\delta}}}_{2}$	5.32978	Fitted
${\varvec{\sigma}}$	≥ 0	Fitted

### Numerical results

We consider the system (1) with newly reported coronavirus cases in Pakistan to obtain the numerical results. Time is defined in days, and parameters values presented in Table 2 are fitted using the nonlinear least-square curve technique. The initial conditions used in the graphical results are $S\left( 0 \right) = 2100000000, I\left( 0 \right) = 9700000, V\left( 0 \right) = 300000, R\left( 0 \right) = 0.$

**Fig. 3:**
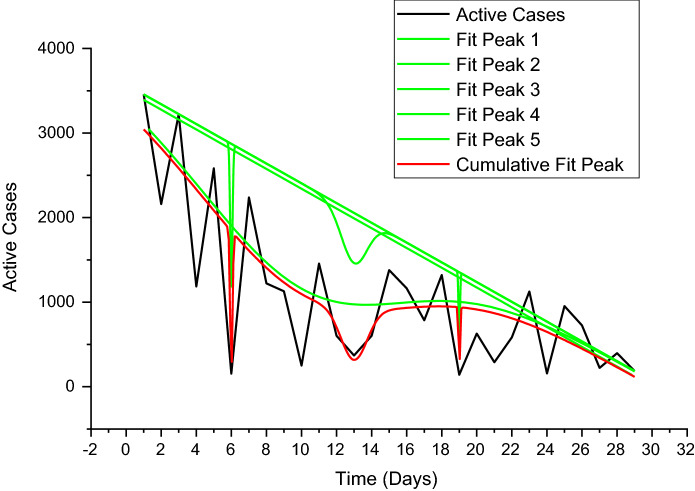
Model prediction to determine the peak of active cases

**Fig. 4:**
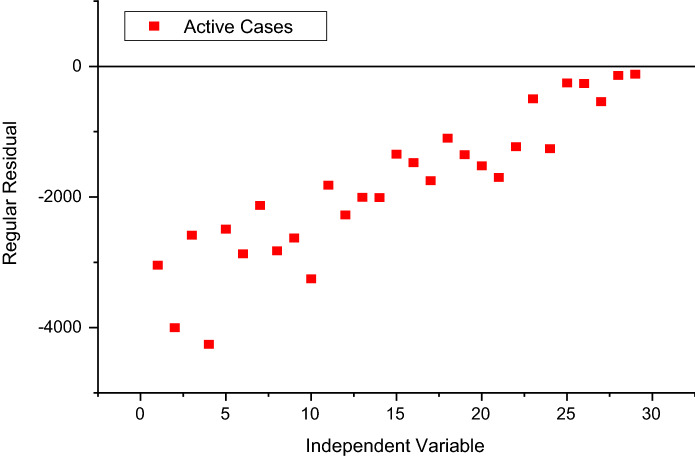
Regular residue of active cases from August 01, 2021, to August 29, 2021

**Fig. 5:**
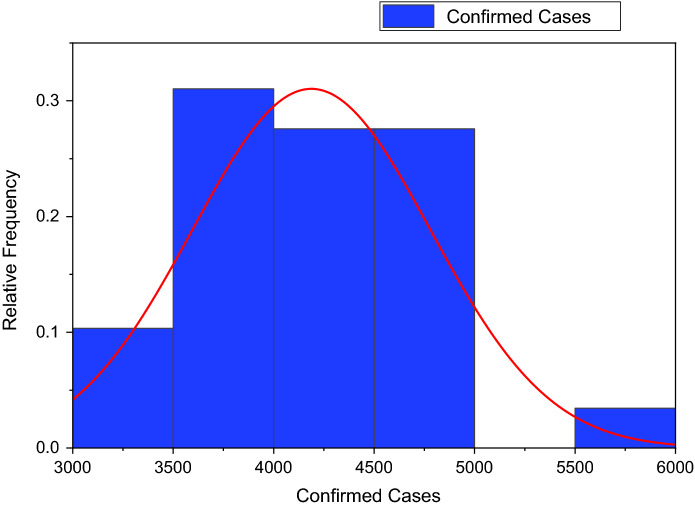
Frequency of confirmed cases from August 01, 2021, to August 29, 2021

**Fig. 6:**
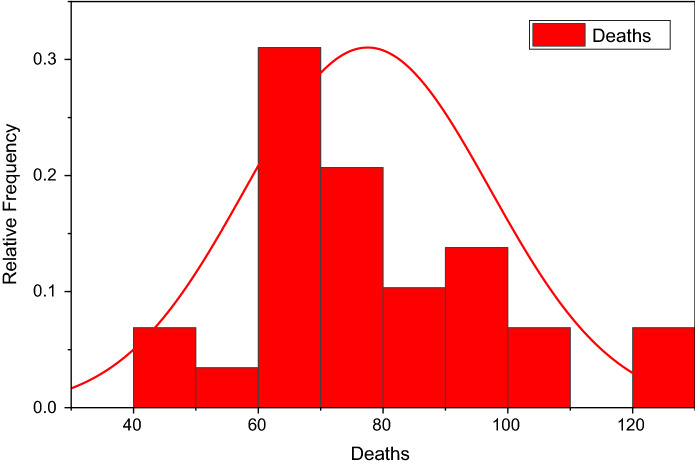
Frequency of deaths from August 01, 2021, to August 29, 2021

**Fig. 7:**
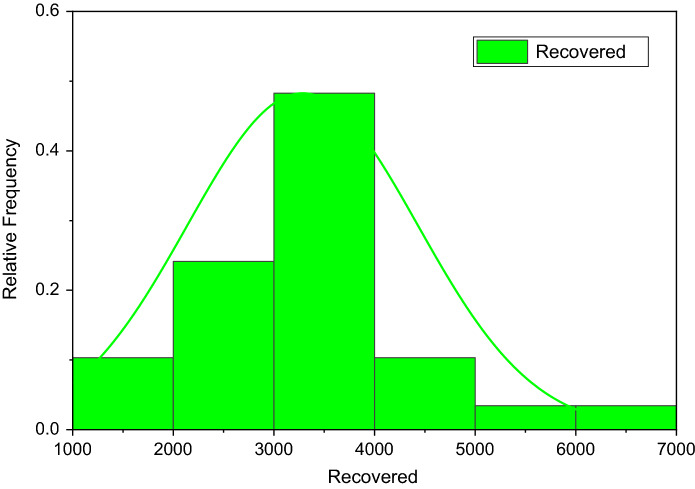
Frequency of recovered cases from August 01, 2021, to August 29, 2021

**Fig. 8:**
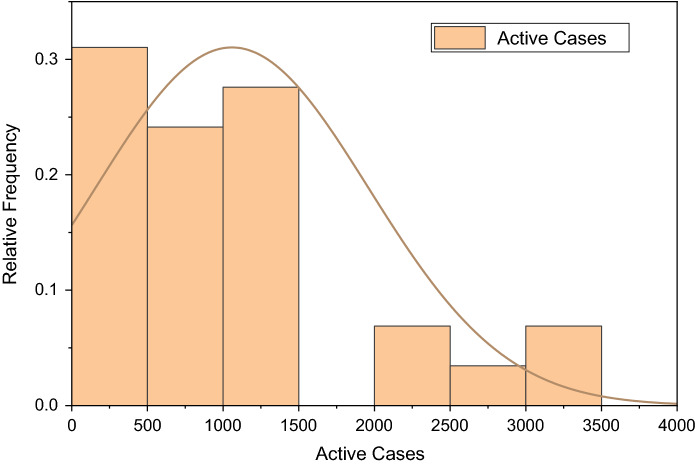
Frequency of active cases from August 01, 2021, to August 29, 2021

**Fig. 9:**
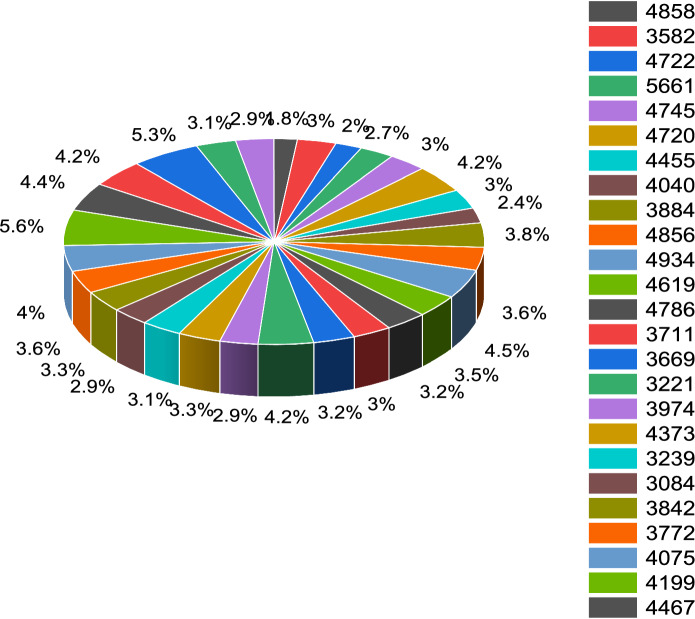
Percentage of confirmed cases in the pie chart

**Fig. 10:**
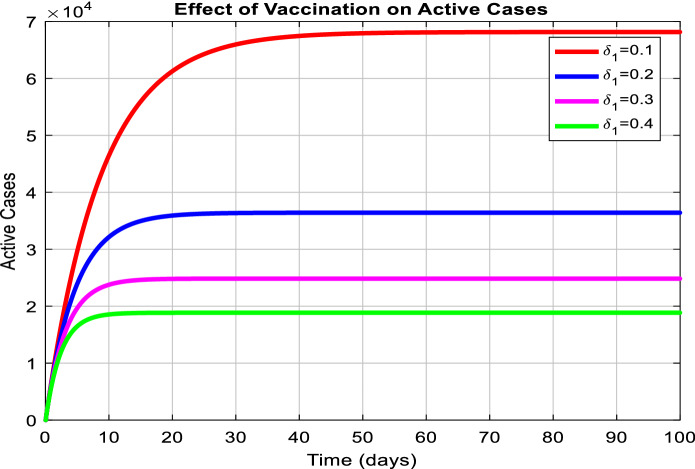
Impact of vaccination on active cases

**Fig. 11:**
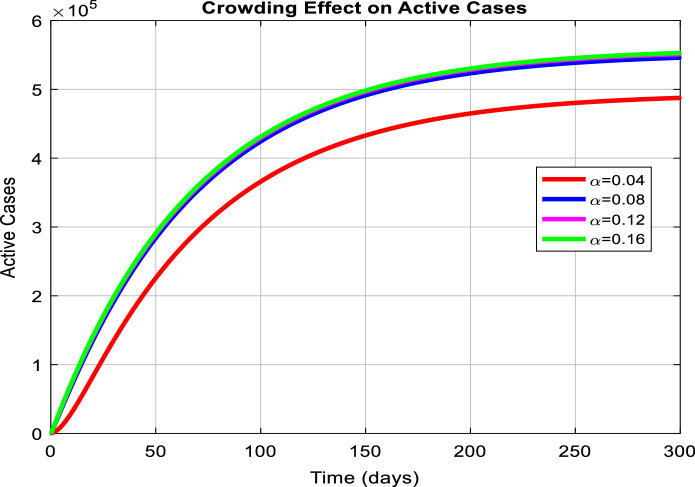
Impact of crowding effect on active cases

Figure 3 depicts the prediction to determine the peak of active cases. Figure 4 illustrates the regular residue of active cases. Figures 5, 6, 7, 8, and 9 outline the frequency of confirmed cases, deaths, recovered active cases, and percentage of the confirmed case in the pie chart. Figure 10 shows that systematic uses of vaccination may control the increase in the strain of coronavirus. On the other hand, Fig. 11 indicates that the crowding effect decreases the impact of vaccination on the population.

### Nonstandard finite difference method

This section aims to provide a nonstandard finite difference discretization of the mathematical model (1). To this effect, we divide the interval [0, L] into M ∈ N subintervals, respectively, and step sizes h = L/M. The approximate solutions $S, I, V, \mathrm{and} R$ of (1) will be denoted as ${S}^{n}, {I}^{n}, {V}^{n}, and {R}^{n}$, respectively, for each $n=\mathrm{0,1}, \dots ,N.$ Under the rules, the discretization of a system (1) is presented in [43]. We have

17 $$ S^{n + 1} = \frac{{S^{n} + h\Lambda }}{{1 + h\frac{{\beta I^{n} }}{{1 + \alpha I^{n} }} + h\left( {\delta_{2} + \mu } \right)}}, $$

18 $$ I^{n + 1} = \frac{{I^{n} + h\frac{{\beta S^{n} I^{n} }}{{1 + \alpha I^{n} }}}}{{1 + h\left( {\gamma + \delta_{1} + \mu } \right)}}, $$

19 $$ V^{n + 1} = \frac{{V^{n} + h\delta_{2} S^{n} + h\delta_{1} I^{n} }}{{1 + h\left( {\sigma + \mu } \right)}}, $$

20 $$ R^{n + 1} = \frac{{R^{n} + h\gamma I^{n} + h\sigma V^{n} }}{1 + h\mu }, $$

**Fig. 12:**
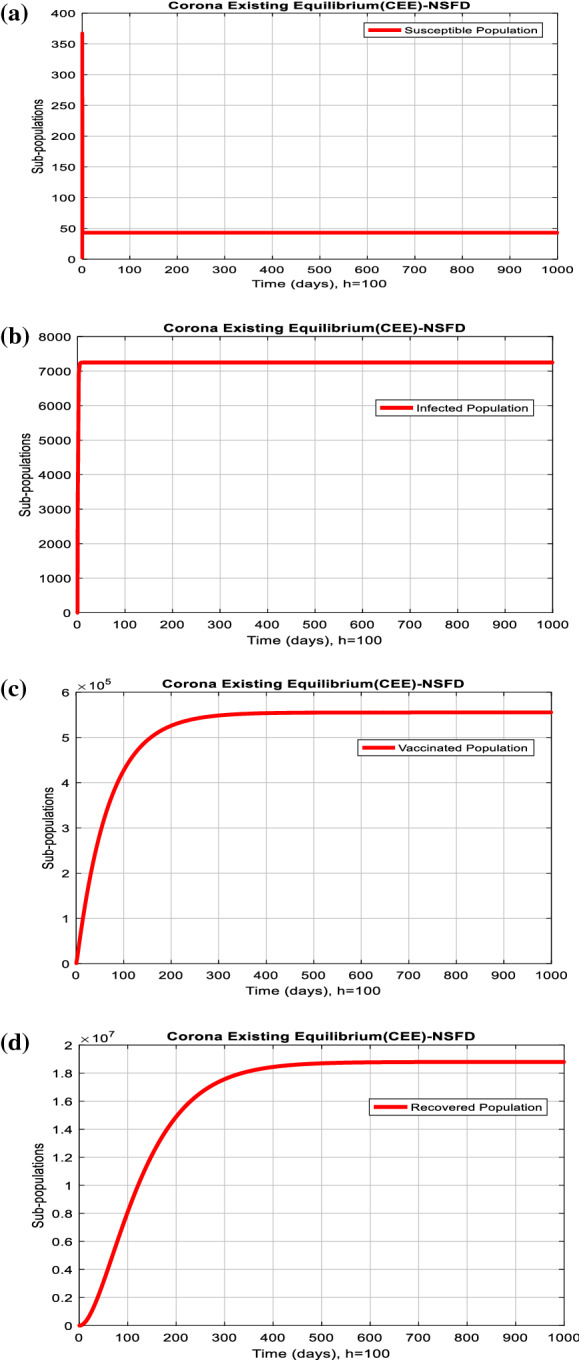
Graphs of (**a**) susceptible, (**b**) infected, (**c**) vaccinated, and (**d**) recovered population versus time t, at the corona existing equilibrium

The simulation of the system (17)–(20) is done by using objective data estimation. It reveals the beauty of this method which is routed for long-term behavioral analysis of the model. Also, the nonstandard finite difference method commits the model's dynamical properties like positivity, boundedness, consistency, and stability, as shown in Fig. 12.

## Concluding remarks

A mathematical model has been developed to study the third strain of coronavirus in Pakistan. The infected cases were taken from August 01, 2021, till August 29, 2021. The model is asymptotically stable in the sense of local (if $${R}_{0}<1)$$ and global (if $${R}_{0}>1)$$ at both equilibria. Moreover, we determine the basic reproduction number, $${R}_{0}$$ using the reported active cases during the stipulated time. The least-square curve fitting method is used to obtain the realistic parameters and the numerical results graphically. The effect of crowding on the dynamics of coronavirus is also shown. In the same way, due to the development in vaccination programs worldwide, it is one of the significant measures taken to overcome the menace of the new strain, and its efficiency is shown graphically using the developed model. Furthermore, we analyzed the graphical results and found that the increase in coronavirus cases of the third strain can effectively be controlled if the SOPs designed by the World Health Organization (WHO) are followed. We also observed that the active cases could be reduced in Pakistan as shown by the results from our model. Knowing the peak of the third strain is vital for any country and determines the maximum active cases on a particular day. These results can be helpful for planning and valuable to the Ministry of Health (MOH) and the decision-making authority: National Command and Operation Center (NCOC) in Pakistan. This type of analysis can be applied to data from neighboring countries such as India, Iran, and Bangladesh.

## Declarations

### Data availability

Data will be available on reasonable request.
